# Evaluation of biological selenium nanoparticles on growth performance, histopathology of vital organs and genotoxicity in Japanese quails (*coturnix coturnix japonica)*

**DOI:** 10.1080/01652176.2024.2319830

**Published:** 2024-04-01

**Authors:** Shabana Naz, Gulnaz Bibi, Rida Nadeem, Ibrahim A. Alhidary, Sifa Dai, Muhammad Israr, Rifat Ullah Khan

**Affiliations:** a Department of Zoology, Government College University, Faisalabad, Pakistan; b Department of Animal Production, College of Food and Agriculture, King Saud University, Riadh, Saudi Arabia; c Department of Pharmaceutical and Life Sciences, Jiujiang University, Jiujiang City, Jingxi province, P.R. China; d College of Veterinary Sciences, The Univeristy of Agriculture, Peshawar, Pakistan

**Keywords:** Comet assay, cytotoxicity, genotoxicity, growth performance, histopathology, Japanese quail, Selenium-nanoparticles

## Abstract

Research on the effects of selenium nanoparticles (Se-NPs), particularly in Japanese quails, is lacking, especially regarding the potential for DNA damage. Therefore, this study aimed to investigate the impact of administering 0.2 and 0.4 mg/kg of Se-NPs on the growth performance, DNA integrity, and histopathological alterations of the liver, lung, kidney, and heart in quails. A total of 480 one-day-old Japanese quails were divided into three experimental groups as follows: Group 1 served as the control and received only basic feed, while Group 2 and 3 received 0.2 mg/kg and 0.4 mg/kg of Se-NPs *via* oral gavage. Our results suggested that, birds fed with Se-NPs at both levels significantly (*p* < .01) reduced feed intake, however, weight gain was significantly (*p* < .01) increased in quails supplemented with 0.2 mg/kg. Similarly, feed conversion ratio (FCR) was significantly (*p* < .01) reduced in group supplemented with 0.2 mg/kg Se-NPs. White blood cells increased significantly (P0.01) in 0.4 mg/kg while haemoglobin and red cell distribution width decreased (*p* < .01) in the same group. Both treatment regimens resulted in DNA damage and histopathological alterations; however, the adverse effects were more prominent in the group receiving the higher dose of 0.4 mg/kg. These findings indicate that the lower dose of 0.2 mg/kg may have beneficial effects on growth. However, the higher dose of 0.4 mg/kg not only negatively impacts growth but also leads to histopathological alterations in major organs of the body and DNA damage as well.

## Introduction

The field of nanotechnology is undergoing rapid expansion and encompasses diverse realms of academic research (Aziz et al. 2022). Its potential for making substantial strides in human and animal health is noteworthy, especially in domains such as bolstering resistance against pathogens, employing antioxidants, degrading toxins, and enhancing nutrient efficiency (Reda et al. 2020, 2021). Selenium, found in organic (seleno-cysteine and seleno-methionine) and inorganic (selenate and selenite) forms, is relatively rare in nature. Human activities, such as refining, fossil fuel combustion, agricultural practices, oil smelting, and industrial mine sewage discharge, have led to significant contamination issues in soils and water bodies (Khan et al. 2022; Hassan et al. 2023). This element bioaccumulates in polluted areas, entering plants, then livestock that consumes these plants, and ultimately reaching humans who consume the animals. Selenium is crucial for various functions in the human body, including metabolism, thyroid hormone regulation, DNA synthesis, reproduction, and protection against oxidative and infectious damage, being a component of seleno-proteins and seleno compounds (Kumar and Prasad 2021; Ostovar et al. 2022).

Poultry feed additives currently encompass inorganic selenium forms like sodium selenite, and organic forms such as selenomethionine, selenocysteine, and Se-enriched yeast. The supplementation of selenium has been shown to enhance the growth performance, antioxidant status and immune system of heat-stressed broiler chickens (Bami et al. 2022). Selenium, a crucial trace element in the diet, plays a vital role in both internal and external organ function, contributing to animal growth, immunity, hormonal balance, and fertility (Kassim et al. 2022). Selenium nanoparticles have demonstrated significantly higher efficiency compared to selenium salts, and they are also less toxic (Chen et al. 2022).

Selenium nanoparticles (Se-NPs) have been successfully synthesized through various green nanotechnology approaches, including the use of plant extracts, as reported by Zambonino et al. (2021) and Medina Cruz et al. (2018). Green synthesis offers an eco-friendly method for nanoparticle synthesis, utilizing both unicellular and multicellular living organisms, such as yeast, fungi, bacteria, plant tissues, and algae, as natural reducing and stabilizing agents (Malyugina et al. 2021). Multiple studies have demonstrated that the form and dosage of selenium in the diet can significantly impact the growth performance, antioxidative properties, immunity and meat quality attributes in broilers. Nevertheless, as per the authors’ knowledge, there is a scarcity of information regarding the utilization of nano-Se in Japanese quails diets. Therefore, the main objective of the present study was to evaluate the impact of different levels of biologically synthesized nanoselenium supplementation at the rate of 0.2 and 0.4 mg/kg on the growth performance, hematological parameters, histopathological changes in vital organs, and DNA damage in Japanese quails.

## Materials and methods

### Extraction of capsicum extract

The contaminants associated with *Capsicum annum* were meticulously removed by washing them with double-distilled water seven times. The cleaned components were then sliced into small pieces and allowed to air-dry in the shade for a period of 15 d. Subsequently, the dried pieces were ground into a powder using a grinder. The resulting 500 g powder of *Capsicum annum* was subjected to a maceration process, wherein it was added to 1000 ml of ethanol. The mixture underwent filtration using filter paper to separate the filtrate. The obtained filtrate was processed in a rotary evaporator for approximately 15 min, and the resulting extract was further treated in a vacuum evaporator until a constant weight was achieved. This process yielded a dry cake extract.

### Synthesis of selenium nanoparticles (Se-NPs)

For the synthesis of selenium nanoparticles (Se-NPs), 50 ml of the *Capsicum annum* extract was mixed with 0.263 g of selenious acid (H_2_SeO_3_). Over a 15-h period, the pH of the mixture was maintained at 5.4 by adding NaOH (sodium hydroxide), resulting in a red color appearance (NaOH was added to adjust the pH from 4.8). The mixture was then centrifuged at 12,000 rpm for 4 min at room temperature. The deposited material was washed with deionized water and left in tubes for complete drying before being weighed (Batra et al. 2017).

### Birds, experimental design and management

A total of 480 one-day-old *C. coturnix japonica* chicks were randomly allocated into three groups, each consisting of eight replicates. Group 1 served as the control group, receiving a normal basal diet. Groups 2 and 3 were supplemented with 0.2 and 0.4 mg/kg respectively. The quails were housed in wire cages under consistent housing and management conditions, adhering to standard hygiene protocols for a 10-day acclimatization period. The temperature was maintained at approximately 36 °C, and humidity ranged from 50 to 70%. Throughout the trial, the birds were provided with commercial broiler feed (Table 1). They had unrestricted access to water and food, and their light cycle consisted of 18 h of sunlight and 6 h of darkness. All groups followed identical lighting, feeding, and watering procedures throughout the experiment.

**Table 1.** Feed ingredients and nutrient content of the basal diets.

Ingredients	g/100 g	Chemical composition	g/100 g
Corn	54.0	Metabolizable energy, kcal/kg	2902
Soybean meal (46 % CP)	27.0	Crude protein	20.1
Sunflower meal (30 % CP)	7.0	Crude fat	7.3
Soybean oil	4.3	Crude fiber	4.1
Calcium carbonate	5.6	Moisture	12.7
Dicalcium phosphate	1.2	Calcium	2.5
Salt	0.4	Available phosphorus	0.4
Premix	0.3	Lysine	1.0
DL methionine	0.2	Methionine	0.5
		Cystine	0.4
		Methionine + cysteine	0.8
		Selenium, mg/kg	0.1
Total	100.00		

### Growth performance

The body weight gain (BWG) and feed intake of the birds for the experimental period (1–35 d) was calculated on weekly basis. The obtained data was used to calculate FCR. Mortality was recorded as occurred (Foroutankhah et al. 2019).

### Haemotological analysis

At the 35th day of the study, blood samples were obtained from 3 chicks per replicate through cardiac puncture using EDTA tubes. The collected blood samples with EDTA were carefully placed into properly labeled and sterilized tubes, specifically designated for the analysis of hematological parameters. Hematological parameters include lymphocytes, white blood cells, mid-range, red blood cells, granulocytes, haemoglobin, mean corpuscular volume, haematocrit, platelet count, mean corpuscular haemoglobin and mean corpuscular hemoglobin concentration.

### Histopathological examination

At the conclusion of the study, three birds per replicate were slaughtered, and their skin was removed. Liver, heart, and kidneys were then aseptically extracted. These tissues were preserved in a 10% formalin neutral solution for subsequent evaluation of potential histopathological alterations. Following preservation, the tissues were processed and sliced to approximately 4 μm thickness. Finally, the tissues were stained with hematoxylin-eosin according to the method outlined by Landy et al. (2020).

### Comet assay

DNA damage in japanese quails was assessed using the Comet assay following the protocol outlined by Singh et al. (1988) with slight modifications. Ten samples from each group were analyzed using the Comet assay technique.

### Statistical analysis

The data were processed using Microsoft Excel (USA) and Statistica version 12.0 (CZ). Statistical analysis was performed using a one-way analysis of variance (ANOVA) with sex as a covariate can be used to adjust for any potential differences between male and female quails. Scheffe’s test was then applied to determine significant differences with confidence. A significance level of *p* < .05 was used to identify statistically meaningful distinctions between the groups.

## Results

Table 2 displays the weekly feed intake of *C. coturnix japonica* under various Se-NPs treatments. The findings indicated that at 0.4 mg/kg Se-NPs, the feed intake was significantly (*p* < .01) lower compared to both 0.2 mg/kg and the control group on weekly basis except week 1.

**Table 2.** Feed intake, weight gain and feed conversion ratio in Japanese quails supplemented with different levels of selenium nanoparticle.

Weeks	Control	0.2 mg/kg	0.4 mg/kg	*p* value
**Feed intake (g)**				
Week 1	44.61 ± 0.05	42.66 ± 0.01	41.20 ± 0.25	.11
Week 2	62.40 ± 0.10^a^	53.75 ± 0.01^b^	54.22 ± 0.01^b^	.01
Week 3	83.78 ± 0.11^a^	68.81 ± 0.10^c^	75.06 ± 0.04^b^	.01
Week 4	110.56 ± 0.15^a^	100.70 ± 0.10^b^	98.80 ± 0.10^b^	.01
Week 5	136.65 ± 0.14^a^	135.32 ± 0.11^a^	132.39 ± 0.05^b^	.01
LinearQuadratic	0.01	0.01	0.01	
	0.01	0.01	0.01	
**Weight gain (g)**				
Week 1	26.23 ± 3.677	25.30 ± 3.301	25.15 ± 5.39	.11
Week 2	39.46 ± 8.322	41.76 ± 5.293	40.38 ± 8.16	.11
Week 3	58.15 ± 13.631^c^	64.53 ± 7.523^a^	61.30 ± 11.75^b^	.01
Week 4	83.30 ± 14.682^c^	96.00 ± 9.327^a^	88.76 ± 17.41^b^	.01
Week 5	113.76 ± 16.528^c^	131.38 ± 9.385^a^	121.46 ± 13.76^b^	.01
Linear	0.01	0.01	0.01	
Quadratic	0.01	0.01	0.01	
**Feed conversion ratio (g/g)**				
Week 1	1.7±.01^a^	1.68±.01^a^	1.63±.01^b^	.01
Week 2	1.58±.01^a^	1.28±.01^c^	1.34±.01^b^	.01
Week 3	1.44±.01^a^	1.06±.01^c^	1.22±.01^b^	.01
Week 4	1.31±.01^a^	1.05±.01^c^	1.11±.01^b^	.01
Week 5	1.21±.01^a^	1.03±.01^c^	1.09±.01^b^	.01
0.01	0.01	0.01		
Linear	0.01	0.01	0.01	
Quadratic	0.01	0.01	0.01	

Mean values bearing different superscripts differ significantly (*p* < .01).

Table 2 presents the weekly body weight of *C. coturnix japonica* under different treatments of Se-NPs. Weight gain was significantly (*p* < .01) higher in quails, except in the first and second weeks, when supplemented with 0.2 mg/kg Se-NPs, compared to the 0.4 mg/kg and control groups.

Table 2 displays the weekly Feed Conversion Ratio (FCR) of *C. coturnix japonica* under different treatments of Se-NPs. FCR was significantly (*p* < .01) lower in quails that received 0.2 mg/kg Se-NPs compared to the control and those supplemented with 0.4 mg/kg selenium nanoparticles. Linear and quadratic effects were recorded for feed intake, weight gain and FCR for different Se-NP compared to the control.

Table 3 displays the cell Blood Count (CBC) of *C. coturnix japonica* reared under control and Se-NPs supplementation. The results indicated that WBC count was significantly (*p* < .01) higher in quails supplemented with 0.2 mg/kg selenium nanoparticles compared to 0.4 mg/kg and the control group. However, lymphocyte percentage decreased significantly (*p* < .01) in the treatment group compared to the control. Hemoglobin concentration decreased significantly (*p* < .01) in the 0.4 mg/kg supplemented selenium nanoparticles group. All other parameters did not change significantly between the control and the treatment groups. Linear and quadratic effects were seen for WBCs, lymphocytes and red cell distribution width in the treatment groups compared to the control.

**Table 3.** Complete blood count of Japanese quails supplemented with different levels of selenium nanoparticles.

Parameters	Control	0.2 mg/kg	0.4 mg/kg	*p*-value	Orthogonal contrast
White blood cells (10^3^/μl)	102.20 ± 4.89^b^	137.10 ± 0.687^a^	123.62 ± 1.46^a^	.001	Linear: 0.01 Quadratic: 0.01
Lymphocyte (%)	36.08 ± 0.45^a^	38.48 ± 0.36^b^	38.56 ± 0.35^b^	.001	Linear: 0.01 Quadratic: 0.01
Mid range (%)	1.34 ± 0.05	1.18 ± 0.06	1.02 ± 0.080	.118	
Granulocytes (%)	1.26 ± 0.051	1.24 ± 0.05	1.34 ± 0.051	.301	
Hemoglobin (g/dl)	22.42 ± 0.36^a^	23.62 ± 0.62^a^	18.28 ± 0.50^b^	.001	
Mean corpuscular hemoglobin (pg)	84.90 ± 0.49	78.56 ± 0.31	82.14 ± 0.95	.11	
mean corpuscular hemoglobin concentration (%)	51.72 ± 0.59	49.04 ± 0.45	50.52 ± 0.36	.17	
Mean corpuscular volume (fl)	160.22 ± 0.99	158.38 ± 0.73	166.84 ± 0.71	.101	
Red blood cells (10^6^/μl)	2.63 ± 0.042	3.06 ± 0.033	2.31 ± 0.063	.21	
Hematocrit (%)	33.50 ± 0.49	37.94 ± 0.66	36.12 ± 1.01	.31	
Red cell distribution width (%)	18.54 ± 0.47^a^	15.06 ± 0.66^b^	12.92 ± 0.44^c^	.01	Linear: 0.01 Quadratic: 0.01
Platelets (10^3^/μl)	2.60 ± 0.51	2.63 ± 0.510	2.80 ± 0.374	.12	

Mean values bearing different superscripts differ significantly (*p* < .01).

Table 4 showed various comet parameters observed in the blood of C. coturnix japonica in response to different doses of Se-NPs. The results indicate that L tail, L comet, tail DNA, TM, and OTM increased significantly (*p* < .001) in quails supplemented with 0.4 ppm selenium nanoparticles. However, head DNA decreased significantly (*p* < .001) in the higher dose (0.4 ppm) of selenium nanoparticles. The orthogonal results also showed linear and quadratic effects of the Se-NPs in comparison to the control.

**Table 4.** Comet parameters observed in blood of Japanese quails supplemented with different levels of selenium nanoparticles.

	Treatments				
Comet parameters	Control	0.2 mg/kg	0.4 mg/kg	*p*-value	Orthogonal contrast
Head length	22.800 ± 2.032	19.600 ± 1.551	19.800 ± 2.313	.455	–
Tail length	3.900 ± 0.277^c^	9.600 ± 0.581^b^	21.000 ± 1.308^a^	.001	Linear: 0.01 Quadratic: 0.01
Comet length	26.333 ± 2.242^b^	29.200 ± 1.825^b^	40.800 ± 2.851^a^	.001	Linear: 0.01 Quadratic: 0.01
Head DNA	97.355 ± 0.661^a^	91.098 ± 1.517^b^	71.997 ± 2.634^c^	.001	Linear: 0.01 Quadratic: 0.01
Tail DNA	2.645 ± 0.661^c^	8.902 ± 1.517^b^	28.003 ± 2.634^a^	.001	Linear: 0.01 Quadratic: 0.01
Tail moment	0.105 ± 0.0276^b^	0.873 ± 0.158^b^	5.766 ± 0.512^a^	.001	Linear: 0.01 Quadratic: 0.01
Olive tail moment	0.299 ± 0.0578^c^	1.109 ± 0.144^b^	4.281 ± 0.377^a^	.001	Linear: 0.01 Quadratic: 0.01

Mean values bearing different superscripts differ significantly (*p* < .01).

L-Head = Head Length; L-Tail = Tail Length; L-Comet = Comet Length; % DNA Head = Head % DNA Tail = DNA Percent, Tail DNA Percent; TM = Tail Moment; OTM = Olive Tail Moment.

### Histological analysis of liver

**Figure 1.**
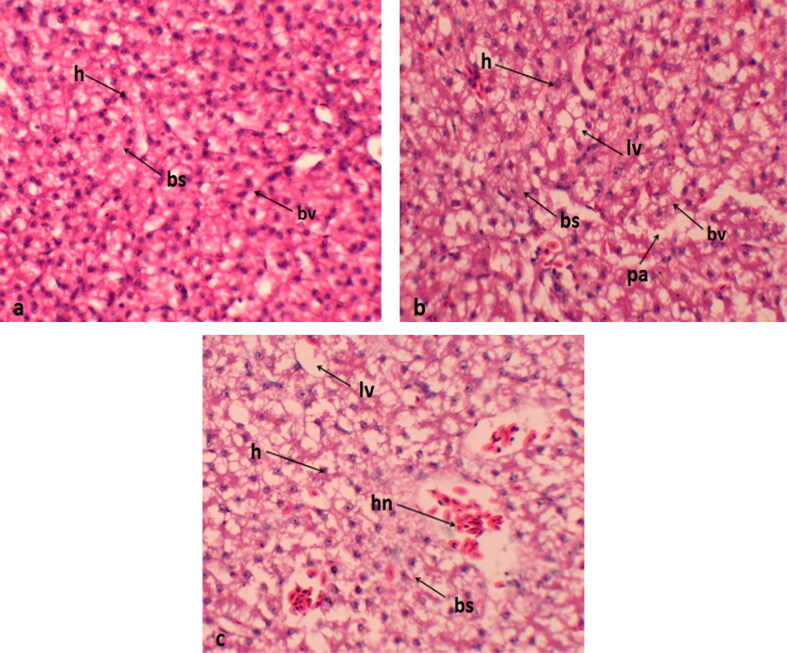
Histological sections of liver (a) normal histological features; (b) dilation of sinusoids and mild vacuolization of hepatocellular cytoplasm in 0.2 mg/kg group; (c) Quails received 0.4 mg/kg Se-NPs showing numerous necrotic hepatocytes, bs: blood sinusoid; h: hepatocyte; lv: lipid vacuole; bv: blood vessels; hn: hepatocyte necrosis.

Histological sections of the liver from the control group displayed normal histological architecture. In contrast, the liver from birds supplemented with 0.4 mg/kg exhibited blood sinusoid dilation and mild hepatocellular cytoplasmic vacuolization (Figure 1).

### Histological analysis of heart

**Figure 2.**
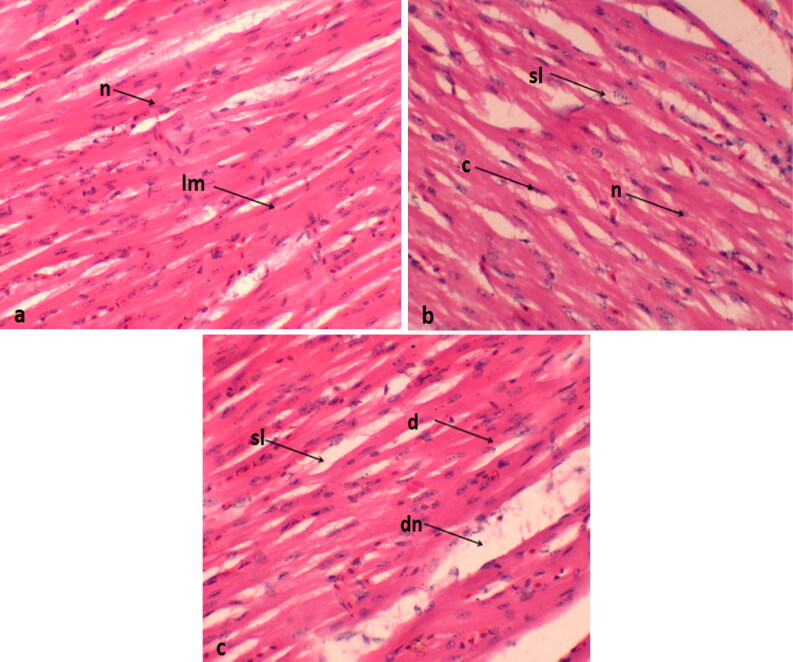
Histological sections of the heart of *coturnix coturnix japonica* treatments are (a) control; (b) 0.2 mg/kg group; (c) 0.4 mg/kg. lm: longitudinal muscles; n: normal nucleus; sl: splitting of muscles; c: congestion; d: degeneration; dn: dislocation of nucleus.

In the control group, myocardial fibers exhibited regular organization, normal nuclei, and a well-arranged pattern with distinct borders. However, the treatment group exposed to 0.4 mg/kg displayed muscle splitting and nuclei dislocation (Figure 2).

### Histological analysis of kidney

**Figure 3.**
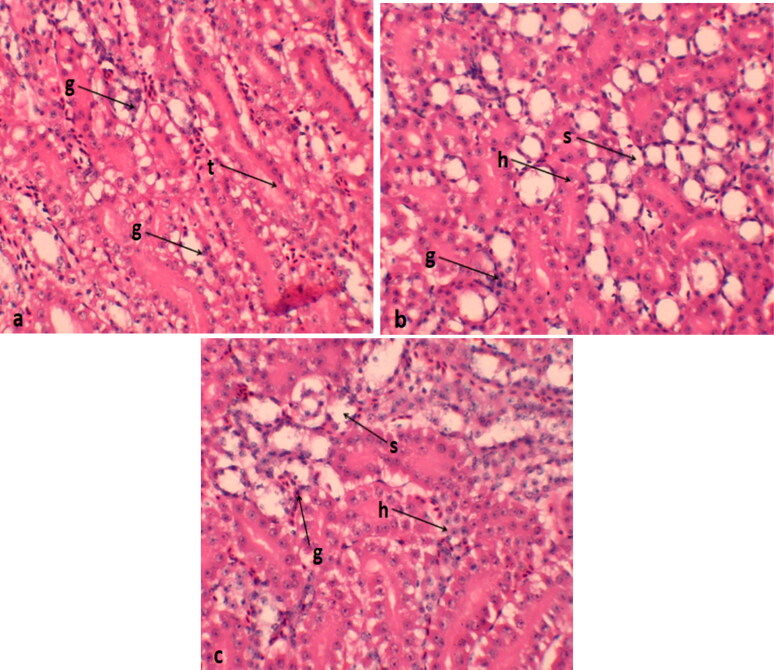
Histological features of kidney of Japanese quails supplemented with selenium nanoparticles (a) control group; (b) Se-NP treatment at the rate of 0.2 mg/kg; (c) Se-NP treatment at the rate of 0.4 mg/kg. g: glomerular; t: renal tubules; s: swelling; h: hemorrhage; s: sloughing of epithelial cells.

The control group exhibits normal kidney tissue, consisting of glomeruli and renal tubules bordered by simple cuboidal epithelial tissue. However, the majority of the epithelial cells lining the renal tubules in this group showed significant signs of degeneration and swelling. These histological alterations indicated variations in the size of the glomeruli, with the 0.4 mg/kg supplementation displaying more pronounced swelling compared to the 0. 2 mg/kg supplemented quails (Figure 3).

### DNA damage assay

**Figure 4.**
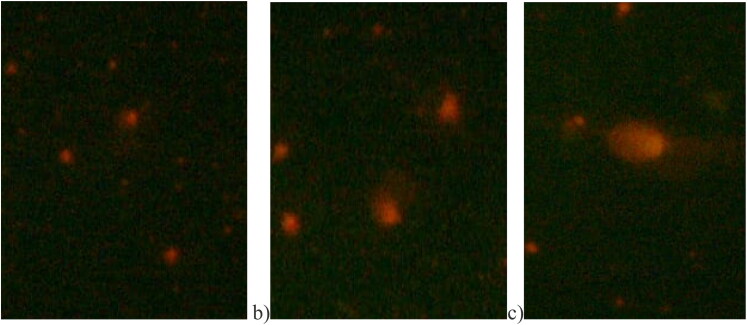
Comparison of DNA damage images of *C. coturnix japonica* exposed to Se-NPs doses (0.2 mg/kg and 0.4 mg/kg). After the comet assay process, pictures were taken at magnification ×30X. (a) Control, normal cell without a tail; (b) 0.2 mg/kg Se-NPs treated group cell showing a tail and a head like a comet; (c) 0.4 mg/kg Se-NPs treated group showing longer tail.

The results from the comet assay are illustrated in Figure 4. In the control group, cells appear normal without a tail. In quails supplemented with 0.2 mg/kg, cells display a distinct Comet-like structure with both a tail and a head. Furthermore, in quails supplemented with 0.4 mg/kg, cells exhibit an even more pronounced Comet tail.

## Discussion

Selenium plays a crucial role as a component of enzymes as a component of antioxidant enzymes to neutralize free radicals generated during oxidation processes, owing to its antioxidant properties. It is a part of unique active proteins called selenoproteins (Kieliszek and Błażejak 2013). Selenium nanoparticles, while having low toxicity, can be damaging even in minimal amounts. In comparison to other selenium species, Se-NPs exhibit potent anticancer efficacy with fewer associated toxicity issues (Ifijen et al. 2023). In the context of current growth performance, especially FCR, Se-NPs at low doses (0.2 mg/kg) have demonstrated a higher growth rate than the control and high-dose groups. Jiang et al. (2009) emphasized selenium’s essential role as a trace element in various physiological processes. These nanoparticles offer enhanced bioavailability, facilitating improved absorption and utilization by quails. Selenium, a crucial component of selenoproteins, influences antioxidant defense mechanisms and supports immune function. It also plays a vital role in thyroid hormone metabolism, impacting overall growth and development. The use of selenium nanoparticles in quail diets has shown promising results in enhancing growth parameters, including weight gain and feed efficiency, highlighting their potential as a nutritional supplement for optimizing the growth performance of Japanese quails. Dawood et al. (2020) found that the inclusion of Se-NPs significantly improved feed conversion ratio and feed intake. Similarly, Ahmadi et al. (2018) demonstrated that diets containing Se-NPs at levels of 0.2 and 0.4 mg/kg improved the development and carcass characteristics of broilers without any negative effects on their internal organs. Eid et al. (2023) illustrated how bird performance may be enhanced by selenium in nano form, attributing these benefits to antioxidant activity, encouraging increased protein production by the bird’s enzymatic system, and consequently improving all aspects of the physiological response.

Concerning the feed intake of quails, the results indicated a significant reduction in total feed intake when quails were fed with different levels of Se-NPs. These findings are consistent with the observations of Eldeeb and Ibrahim (2023), who noted that various levels of Se-NPs at rates of 0.2, 0.4, and 0.6 ppm led to a decrease in feed intake in ducks. Similarly, El-Kazaz et al. (2020) showed in an experiment of Japanese quails that administering 0.2 mg/kg Nano-Se significantly decreased feed intake. Similarly, Jamima et al. (2020) recorded that a dose of 0.15 mg/kg Nano-Se in birds had the lowest feed consumption and improved FCR. Moreover, reduced feed intake in broilers in response to 0.3 mg/kg nano-selenium was also reported by Eid et al. (2022).

The current study revealed a significantly lower FCR in quails supplemented with 0.2 mg/kg Se-NPs. These findings align with the observations of Eldeeb and Ibrahim (2023), who reported improved FCR in ducks with various levels of selenium nanoparticles at rates of 0.2, 0.4, and 0.6 ppm. The advantageous effects of nano selenium on growth traits may be attributed to its inherent chemical as well as physical properties, which are biologically produced. Nanoparticles possess a greater surface area, which increases the available surface area, potentially enhancing mineral digestion, bioavailability, and utilization (Sa’aci et al. 2021). Our results are in agreement with Ahmadi et al. (2018), who found improved FCR in broilers fed a diet enriched with nano-Se (0.3 mg/kg). Likewise, Saleh and Ebeid (2019) discovered that in response to 0.5 mg nano-Se/kg feed enhanced FCR in broilers. Furthermore, Sa’aci et al. (2021) demonstrated that broilers fed diets containing 0.10–0.25 mg of NS exhibited improved FCR. Additionally, Eid et al. (2022) recorded that 0.3 mg/kg nano-selenium exhibited better FCR ccompared to those on the control diet, which had lower FCR values.

Nanoparticles provide an opportunity to provide enhanced bioavailability and therapeutic agents thorough high surface availability and size of the particles (Naz et al. 2024). Experiments have proved that nanoparticles showed better transport, uptake and absorption capacity (Aslam et al. 2023) leading to improved growth performance (Eldeeb and Ibrahim 2023). Different factors such as dose, duration, strain of birds and other experimental conditions may be responsible for the changes in the growth performance.

In terms of the hematological analysis of quails, fed different amounts of Nano-Se, a noteworthy increase was observed in blood lymphocyte and WBCs count a. These findings align with the results reported by Eldeeb and Ibrahim (2023), who observed that different levels of selenium nanoparticles at rates of 0.2, 0.4, and 0.6 ppm increased total leukocyte counts and lymphocytes in ducks.

Leukocytosis was attributed to an inflammatory response in the intestinal tract and bone marrow hyperplasia (Fotouh et al. 2014). In the current study, granulocytes, MCH, MCV, MCHC and platelets did not change significantly between the control and the treatment groups. In the same vain, Eldeeb and Ibrahim (2023) reported that platelets count, MCHC, MCH, MCV and Hct, did not change significantly between the control and the selenium nanoparticles treated ducks. These findings align with those reported by Boostani et al. (2015), reported that in response to 0.3 mg/kg of Nano-Se had no significant impact on TEC, and PCV in broilers. Similarly, Mohamed et al. (2016) demonstrated in Sinai chick that incorporating nanoselenium did not affect eosinophils and monocytes. In another study, Alagawany et al. (2021) determined that supplementation different doses of Se-NP rangning from 0.2 to 0.6 g/kg had no influence on granulocytes, RBCs, hematocrit, and MCV in growing quail. Conversely, Jamima et al. (2020) reported that a dose of 0.15 mg/kg Nano-Se significantly elevated levels of Hb, TEC, and PCV, while the effects on WBCs count was not affected significantly. Furthermore, Eid et al. (2022) demonstrated that dose of Se-NPs at dose rate of 0.3 mg/kg in broiler diets had no significant impact on hematological parameters compared to the control group.

In the current study, histopathological examinations were employed to investigate the cytotoxic effects of Se-NPs on various tissues. The examinations revealed dose-dependent pathological alterations in all examined organs, including the lungs, heart, liver, and kidneys, in groups that received Se-NPs at either 0.2 or 0.4 mg per kilogram of body weight. The most noticeable lesions included cellular cytoplasmic vacuolization, dislocation of the nucleus, swelling, degeneration, necrosis, muscle splitting, hemorrhage, congestion, and a mild to moderate degree of inflammation. Similar results were observed by Akhtar et al. (2016) and Khalaf et al. (2017), who found that NPs induce inflammation, oxidative stress, and consequent damage to proteins, cellular and cytoplasmic organelle membranes, and DNA structures. The visible clinical damages in tissues might be connected to oxidative stress generated by NPs, as well as the associated increase in Se levels, which are considered hazardous to cells and tissues. Lin et al. (2009) and Yang et al. (2009) suggested that the primary mechanism underlying the toxicity of nanoparticles (NPs) is their ability to generate free radicals. Our research findings also support a significant adverse effect of Se-NPs on the major organs of Japanese quails, which could be linked the toxic effects of Se-NPs in our study and closely associated with the increasing levels of deposition of Se in these tissues. Previous research has concluded that NPs dissociate in the acidic environment of the stomach but remain unaffected in the neutral pH of the small intestine (Dang et al. 2021). Within the small intestine, NPs accumulate in the villi, disperse into the bloodstream, and subsequently distribute to various organs, thereby inducing cytotoxicities (De Jong et al. 2019).

Current pathological investigations into the genotoxicity of Se-NPs, validated by a comet assay, revealed higher DNA damage in the groups that received 0.4 ppm Se-NPs compared to the control group. Previous research has indicated that Se-NPs can impact cell viability, oxidative equilibrium, and DNA integrity (Karlsson 2010; Wang et al. 2011). Monteith and Skaar (2021) demonstrated that selenium nanoparticles might interfere with DNA repair mechanisms, leading to the consequnces of DNA damage. This results in base lesions and breakage of DNA strands through reactions with both deoxyribose sugars and the nucleobases of DNA. Additionally, ROS oxidizes DNA, and Se hinders DNA repair and transcriptional regulation, posing a threat to the stability of genetic information. These findings align with other studies in chickens (Morsy et al. 2014), fish (Kousar and Javed 2015; Nikdehghan et al. 2018), and birds (Hussain et al. 2011; Auon et al. 2014).

## Conclusion

The study revealed improved body weight gain and feed conversion ratio (FCR) in Japanese quails administered with 0.2 mg/kg of Se-NPs. However, a higher dose (0.4 mg/kg) led to reduced growth performance, accompanied by significant histological damage and genotoxic effects.

## Data Availability

On request.
